# Supplementary material for: Neurophysiological trajectories in Alzheimer’s disease progression
Source: eLife. 2024 Mar 28;12:RP91044. doi: 10.7554/eLife.91044 (PMC10977971; doi:10.7554/eLife.91044)
Supplement: Supplementary file 1. [file elife-91044-supp1.docx]

**Demographics and Neuropsychological assessments**.

Variable Controls (*𝑛*= 70) Patients with AD (*𝑛*= 78)
Age (years) 70*.*5 ± 8*.*28 63*.*9 ± 8*.*93
Sex (% female) 41 (59%) 50 (64%)
Handedness (% right) 54 (77%) 65 (83%)
Education (years) 17*.*43 ± 1*.*97 16*.*6 ± 2*.*47
MMSE 29*.*36 ± 1*.*00 22*.*67 ± 4*.*79
CDR 0*.*00 ± 0*.*00 0*.*83 ± 0*.*46
CDR-SOB 0*.*03 ± 0*.*12 4*.*32 ± 3*.*13
Modiﬁed trails 0*.*65 ± 0*.*25 0*.*24 ± 0*.*21
Design ﬂuency 12*.*82 ± 3*.*56 6*.*33 ± 3*.*74
Phonemic ﬂuency 17*.*52 ± 5*.*40 10*.*75 ± 5*.*14
Category ﬂuency 23*.*93 ± 4*.*88 12*.*14 ± 6*.*29
Digit span forwards 7*.*16 ± 1*.*34 5*.*04 ± 1*.*52
Digit span backwards 5*.*75 ± 1*.*32 3*.*51 ± 1*.*30
Processing speed 86*.*72 ± 15*.*51 52*.*09 ± 24*.*08
Stroop inhibition 52*.*06 ± 14*.*07 23*.*59 ± 16*.*03
Modiﬁed Rey copy 15*.*50 ± 0*.*77 11*.*55 ± 5*.*01
VOSP number location 9*.*13 ± 1*.*23 6*.*97 ± 2*.*70
Repetition 4*.*83 ± 0*.*42 3*.*52 ± 1*.*41
CVLT learning (%) 66*.*31 ± 13*.*34 38*.*93 ± 14*.*69
CVLT short delay recall (%) 74*.*53 ± 17*.*89 43*.*85 ± 27*.*33
CVLT long delay recall (%) 77*.*84 ± 18*.*05 27*.*56 ± 31*.*07
Modiﬁed Rey recall 12*.*12 ± 2*.*79 5*.*03 ± 4*.*36
Geriatric Depression Scale 2*.*62 ± 2*.*58 6*.*85 ± 4*.*46

• Mini Mental State Examination (MMSE) (score out of 30).

• Clinical Dementia Rating (CDR) (score out of 0, 0.5, 1, 2, 3).

• CDR sum of boxes (CDR-SOB) (score out of 18).
• Modiﬁed trails assess set-shifting and are indicated by the number of correct lines drawn within 60 seconds in the modiﬁed trail making test, which requires the subject to serially alternate between numbers and days of the week.
• Design ﬂuency, assessed using the ﬁlled-dots condition from the design ﬂuency subscale of the Delis-Kaplan Executive Function Scale (DKEFS), was scored as the number of correct designs generated within 60 seconds.
• Category ﬂuency indicates the number of animals listed within 60 seconds.
• Phonemic ﬂuency indicates the number of words starting from the letter ‘D’ listed within 60 seconds.
• Digit span forwards assess auditory attention and are indicated by the number of digits correctly repeated in the same order from a list read by the examiner.
• Digit span backwards assess the verbal working memory and are indicated by the number of digits correctly repeated backwards from a list read by the examiner.
• Processing speed is the number of words correctly read in the congruent Stroop test within 60 seconds.

• Stroop inhibition is the number of words correctly read in the incongruent Stroop test within 60 seconds.
• Modiﬁed Rey copy assesses visual construction copy of the Benson ﬁgure (score out of 17).
• VOSP number location is assessed by the number location task of the Visual Object and Space Perception Battery (score out of 10).
• Repetition is assessed by participants repeating ﬁve phonemically complex sentences following the examiner (score out of 5).
• CVLT leaning indicates the cumulative number of words recalled from trails 1-5 in 16 item CVLT word list (score out of 80) for controls and 9 item CVLT word list (total out of 45) for AD.
• CVLT short delay recall indicates the number of words recalled after a 30 second delay from 16 item CVLT word list (score out of 16) for controls and from 9 item CVLT word list (score out of 9 for AD).
• CVLT long delay recall indicates the number of words recalled after a 10-minute delay from 16 item CVLT word list (score out of 16) for controls and from 9 item CVLT word list (score out of 9 for AD).
• Modiﬁed Ray recall is construction of Benson ﬁgure from memory after 10 minutes (score out of 17).

• Geriatric Depression Scale (GDS) (score out of 30).
