# Supplementary material for: Neurophysiological trajectories in Alzheimer’s disease progression
Source: eLife. 2024 Mar 28;12:RP91044. doi: 10.7554/eLife.91044 (PMC10977971; doi:10.7554/eLife.91044)
Supplement: Supplementary file 2. [file elife-91044-supp2.docx]

**The 94 cortical/subcortical anatomical regions included in the AAL3 atlas**.

No. (left, right) Anatomical description Abbreviation

1, 2 Precentral gyrus PreCG
3, 4 Superior frontal gyrus-dorsolateral SFG
5, 6 Middle frontal gyrus MFG
7, 8 Inferior frontal gyrus-opercular part IFGoper

9, 10 Inferior frontal gyrus-triangular part IFGtria
11, 12 IFG pars orbitalis IFGorb
13, 14 Rolandic operculum ROL

15, 16 Supplementary motor area SMA
17, 18 Olfactory cortex OLF
19, 20 Superior frontal gyrus-medial SFGmed

21, 22 Superior frontal gyrus-medial orbital PFCvent
23, 24 Gyrus rectus REC
25, 26 Medial orbital gyrus OFCmed
27, 28 Anterior orbital gyrus OFCant
29, 30 Posterior orbital gyrus OFCpost
31, 32 Lateral orbital gyrus OFClat
33, 34 Insula INS
35, 36 Anterior cingulate & paracingulate gyri ACC
37, 38 Middle cingulate & paracingulate gyri MCC
39, 40 Posterior cingulate gyrus PCC
41, 42 Hippocampus HIP
43, 44 Parahippocampal gyrus PHG
45, 46 Amygdala AMYG
47, 48 Calcarine ﬁssure and surrounding cortex CAL
49, 50 Cuneus CUN
51, 52 Lingual gyrus LING
53, 54 Superior occipital gyrus SOG
55, 56 Middle occipital gyrus MOG
57, 58 Inferior occipital gyrus IOG
59, 60 Fusiform gyrus FFG
61, 62 Postcentral gyrus PoCG
63, 64 Superior parietal gyrus SPG
65, 66 Inferior parietal gyrus IPG
67, 68 SupraMarginal gyrus SMG
69, 70 Angular gyrus ANG
71, 72 Precuneus PCUN
73, 74 Paracentral lobule PCL
75, 76 Caudate nucleus CAU
77, 78 Lenticular nucleus-Putamen PUT
79, 80 Lenticular nucleus-Pallidum PAL
81, 82 Thalamus THA
83, 84 Heschls gyrus HES
85, 86 Superior temporal gyrus STG
87, 88 Temporal pole: superior temporal gyrus TPOsup
89, 90 Middle temporal gyrus MTG
91, 92 Temporal pole: middle temporal gyrus TPOmid
93, 94 Inferior temporal gyrus ITG
