# Supplementary material for: Neurophysiological trajectories in Alzheimer’s disease progression
Source: eLife. 2024 Mar 28;12:RP91044. doi: 10.7554/eLife.91044 (PMC10977971; doi:10.7554/eLife.91044)
Supplement: Supplementary file 3. [file elife-91044-supp3.docx]

**Direct evaluation of the data likelihoods for all possible** *𝑧***-score event sequences in the AC-EBM.** In this example, we select the optimal set of *𝑧*-score events; *𝑧*_PHG_ = [E1 E2 E3] = [0*.*0964 0*.*4708 2*.*4519] and *𝑧*_MMSE_ = [E4 E5 E6] = [0*.*5109 4*.*4116 6*.*2417] (see Step 2 in Figure 1—ﬁgure supplement 1). In an AC-EBM, there are total 20 ordered arrangements of the *𝑧*-score events. This evaluation indicates that the data likelihood, log *𝑃*(*𝑍*|*𝑆*), is maximized when *𝑆*= [E1 E2 E4 E5 E6 E3] (labeled ∗ in the table), as shown in Step 2 in Figure 1—ﬁgure supplement 1. The second highest likelihood was obtained when *𝑆*= [E1 E4 E2 E5 E6 E3] (labeled ∗ ∗ in the table). It is worth noting that *𝑃_**_* /*𝑃*_∗_ ~2.66E-07. This indicates that it is extremely rare for sequences other than the most likely sequence (*𝑆*= [E1 E2 E4 E5 E6 E3]) to occur in the MCMC sampling. This is the reason why we obtained the positional variance diagram without positional uncertainty for the optimal set of *𝑧*-score events in the AC-EBM.

Sequence *𝑆* log *𝑃*(*𝑍*|*𝑆*)
E1 E2 E3 E4 E5 E6 -473.08
E1 E2 E4 E3 E5 E6 -435.16
E1 E2 E4 E5 E3 E6 -407.33

∗ E1 E2 E4 E5 E6 E3 -353.69
E1 E4 E2 E3 E5 E6 -430.07
E1 E4 E2 E5 E3 E6 -417.75

∗∗ E1 E4 E2 E5 E6 E3 -368.83
E1 E4 E5 E2 E3 E6 -434.35
E1 E4 E5 E2 E6 E3 -396.72
E1 E4 E5 E6 E2 E3 -423.01
E4 E1 E2 E3 E5 E6 -462.35
E4 E1 E2 E5 E3 E6 -461.09
E4 E1 E2 E5 E6 E3 -412.08
E4 E1 E5 E2 E3 E6 -487.32
E4 E1 E5 E2 E6 E3 -449.11
E4 E1 E5 E6 E2 E3 -473.24
E4 E5 E1 E2 E3 E6 -545.23
E4 E5 E1 E2 E6 E3 -512.36
E4 E5 E1 E6 E2 E3 -540.27
E4 E5 E6 E1 E2 E3 -596.36
