# Supplementary material for: Neurophysiological trajectories in Alzheimer’s disease progression
Source: eLife. 2024 Mar 28;12:RP91044. doi: 10.7554/eLife.91044 (PMC10977971; doi:10.7554/eLife.91044)
Supplement: Supplementary file 4. [file elife-91044-supp4.docx]

**Top 10 regions with signiﬁcant group differences in GM volumes comparison between AD patients and controls.** Negative *𝑇*-value represents that a mean regional volume in the AD group is smaller than that in controls. The degree of freedom *𝑑𝑓*= 143. A value of 0.000E+00 denotes 2.2204E-16 (double precision).

Regions (AAL3 atlas) *𝑇*-value *𝑝*-value *𝑞*-value Left Inferior temporal gyrus -10.649 0.000E+00 0.000E+00 Left Middle temporal gyrus -10.605 0.000E+00 0.000E+00 Right Middle temporal gyrus -10.253 0.000E+00 0.000E+00 Left Fusiform gyrus -9.758 0.000E+00 0.000E+00 Left Hippocampus -9.457 0.000E+00 0.000E+00 Left Parahippocampal gyrus -8.808 3.997E-15 6.262E-14 Right Inferior temporal gyrus -8.614 1.177E-14 1.580E-13 Left Precuneus -8.585 1.399E-14 1.644E-13 Right Parahippocampal gyrus -8.524 1.976E-14 2.064E-13 Right Hippocampus -8.411 3.775E-14 3.548E-13
