# Supplementary material for: Neurophysiological trajectories in Alzheimer’s disease progression
Source: eLife. 2024 Mar 28;12:RP91044. doi: 10.7554/eLife.91044 (PMC10977971; doi:10.7554/eLife.91044)
Supplement: Supplementary file 5. [file elife-91044-supp5.docx]

**Top** 10 **regions with signiﬁcant group differences in long-range synchrony between patients with AD and controls.** Negative *𝑇*-value represents that a mean regional metric in patients with AD is smaller than that in controls. The degree of freedom *𝑑𝑓*= 145.

Frequency band Regions (AAL3 atlas) *𝑇*-value *𝑝*-value *𝑞*-value

Left Precentral gyrus 3.827 1.925E-04 9.938E-03 Right Superior frontal gyrus-dorsolateral 3.624 4.011E-04 9.938E-03 Right Superior frontal gyrus-medial 3.521 5.741E-04 9.938E-03 Right Anterior cingulate & paracingulate gyri 3.518 5.802E-04 9.938E-03 Left Superior frontal gyrus-medial 3.509 5.993E-04 9.938E-03

delta-theta

alpha

beta

Right Middle frontal gyrus 3.493 6.343E-04 9.938E-03 Right Supplementary motor area 3.425 8.011E-04 1.076E-02 Left Middle frontal gyrus 3.372 9.585E-04 1.092E-02 Left Superior frontal gyrus-dorsolateral 3.346 1.046E-03 1.092E-02 Left Inferior frontal gyrus-opercular part 3.310 1.178E-03 1.107E-02

Left SupraMarginal gyrus -7.638 2.735E-12 2.571E-10 Left Rolandic operculum -6.751 3.280E-10 1.184E-08 Left Middle temporal gyrus -6.665 5.152E-10 1.184E-08 Right Fusiform gyrus -6.651 5.526E-10 1.184E-08 Left Superior temporal gyrus -6.626 6.299E-10 1.184E-08 Left Fusiform gyrus -6.469 1.418E-09 2.222E-08 Left Heschls gyrus -6.391 2.117E-09 2.843E-08 Left Inferior parietal gyrus -6.196 5.679E-09 5.959E-08 Right Hippocampus -6.192 5.800E-09 5.959E-08 Left Thalamus -6.174 6.339E-09 5.959E-08

Right Middle temporal gyrus -8.237 9.459E-14 8.892E-12 Right Angular gyrus -7.553 4.387E-12 2.062E-10 Left Middle temporal gyrus -7.455 7.521E-12 2.301E-10 Left Inferior temporal gyrus -7.406 9.793E-12 2.301E-10 Left Middle occipital gyrus -7.035 7.308E-11 1.160E-09 Left Angular gyrus -7.032 7.404E-11 1.160E-09 Left Superior temporal gyrus -6.817 2.326E-10 3.124E-09 Right Fusiform gyrus -6.647 5.667E-10 6.659E-09 Right Inferior temporal gyrus -6.383 2.208E-09 2.082E-08 Right Inferior parietal gyrus -6.382 2.215E-09 2.082E-08
