# Supplementary material for: Neurophysiological trajectories in Alzheimer’s disease progression
Source: eLife. 2024 Mar 28;12:RP91044. doi: 10.7554/eLife.91044 (PMC10977971; doi:10.7554/eLife.91044)
Supplement: Supplementary file 6. [file elife-91044-supp6.docx]

**Top 10 regions with signiﬁcant group differences in local synchrony between patients with AD and controls.** Negative *𝑇*-value represents that a mean regional metric in patients with AD is smaller than that in controls. The degree of freedom *𝑑𝑓*= 145. A value of 0.000E+00 denotes 2.2204E-16 (double precision).

Frequency band Regions (AAL3 atlas) *𝑇*-value *𝑝*-value *𝑞*-value

Left Inferior temporal gyrus 11.235 0.000E+00 0.000E+00 Right Fusiform gyrus 10.937 0.000E+00 0.000E+00 Right Inferior occipital gyrus 10.655 0.000E+00 0.000E+00 Left Middle temporal gyrus 10.643 0.000E+00 0.000E+00 Left Superior temporal gyrus 10.616 0.000E+00 0.000E+00

delta-theta

alpha

beta

Left Parahippocampal gyrus 10.607 0.000E+00 0.000E+00 Left Heschls gyrus 10.534 0.000E+00 0.000E+00 Left Rolandic operculum 10.521 0.000E+00 0.000E+00 Left Inferior occipital gyrus 10.516 0.000E+00 0.000E+00 Left Fusiform gyrus 10.506 0.000E+00 0.000E+00

Left Inferior temporal gyrus -7.805 1.082E-12 1.017E-10 Left Fusiform gyrus -7.529 4.994E-12 2.347E-10 Left Parahippocampal gyrus -7.327 1.512E-11 4.058E-10 Left Inferior occipital gyrus -7.302 1.727E-11 4.058E-10 Right Amygdala -6.793 2.632E-10 4.949E-09 Right Fusiform gyrus -6.626 6.291E-10 9.856E-09 Right Temporal pole: superior temporal gyrus -6.562 8.771E-10 1.156E-08 Right Inferior occipital gyrus -6.522 1.080E-09 1.156E-08 Right Parahippocampal gyrus -6.517 1.107E-09 1.156E-08 Left Middle temporal gyrus -6.485 1.305E-09 1.227E-08

Left Angular gyrus -8.008 3.468E-13 3.260E-11 Left Superior temporal gyrus -7.747 1.497E-12 7.037E-11 Right Angular gyrus -7.416 9.314E-12 2.918E-10 Right Hippocampus -7.272 2.041E-11 4.795E-10 Left Heschls gyrus -7.194 3.114E-11 5.305E-10 Left Lenticular nucleus-Pallidum -7.178 3.386E-11 5.305E-10 Left Hippocampus -7.089 5.453E-11 7.299E-10 Right Middle occipital gyrus -7.065 6.212E-11 7.299E-10 Right Heschls gyrus -6.998 8.913E-11 9.309E-10 Left Parahippocampal gyrus -6.965 1.058E-10 9.944E-10
