# Supplementary material for: Neurophysiological trajectories in Alzheimer’s disease progression
Source: eLife. 2024 Mar 28;12:RP91044. doi: 10.7554/eLife.91044 (PMC10977971; doi:10.7554/eLife.91044)
Supplement: Supplementary file 7. [file elife-91044-supp7.docx]

**Top** 10 **regions with signiﬁcant weighted mean differences (***𝑞<* 0*.*05**, FDR corrected) in long-range synchrony between stages 4 and 1** **(Figure 2E in the main text)**. The *𝑝*- and *𝑞*-values of 0.000E+00 denote a value less than 1/50*,* 000, where 50*,* 000 is the number of the bootstrap samplings in the non-parametric tests.

Frequency band Regions (AAL3 atlas) *𝛿𝑧* [ Stages 4vs.1] *𝑝*-value *𝑞*-value

Left SupraMarginal gyrus -0.975 2.000E-05 3.760E-04 Left Rolandic operculum -0.933 8.000E-05 9.400E-04 Left Fusiform gyrus -0.911 2.000E-05 3.760E-04 Left Thalamus -0.895 2.000E-05 3.760E-04 Right Temporal pole: middle temporal gyrus -0.878 2.000E-05 3.760E-04

alpha

beta

Left Heschls gyrus -0.856 4.000E-05 6.267E-04 Left Lenticular nucleus-Pallidum -0.839 2.000E-04 1.216E-03 Left Lenticular nucleus-Putamen -0.836 1.200E-04 1.128E-03 Right Inferior temporal gyrus -0.834 3.400E-04 1.522E-03 Left Inferior parietal gyrus -0.833 2.000E-04 1.216E-03

Right Middle temporal gyrus -1.160 0.000E+00 0.000E+00 Left Inferior temporal gyrus -1.016 0.000E+00 0.000E+00 Left Angular gyrus -1.015 0.000E+00 0.000E+00 Left Superior temporal gyrus -0.972 2.000E-05 2.089E-04 Right Inferior temporal gyrus -0.935 0.000E+00 0.000E+00 Right Fusiform gyrus -0.930 2.000E-05 2.089E-04 Left Middle temporal gyrus -0.925 4.000E-05 2.892E-04 Right Superior temporal gyrus -0.924 6.000E-05 3.760E-04 Right Inferior parietal gyrus -0.921 4.000E-05 2.892E-04 Right Angular gyrus -0.917 1.600E-04 5.013E-04
