# Supplementary material for: Neurophysiological trajectories in Alzheimer’s disease progression
Source: eLife. 2024 Mar 28;12:RP91044. doi: 10.7554/eLife.91044 (PMC10977971; doi:10.7554/eLife.91044)
Supplement: Supplementary file 8. [file elife-91044-supp8.docx]

**Top** 10 **regions with signiﬁcant weighted mean differences (***𝑞<* 0*.*05**, FDR corrected) in local synchrony between stages 4 and 1** **(Figure 2F in the main text)**. The *𝑝*-and *𝑞*-values of 0.000E+00 denote a value less than 1/50*,* 000, where 50*,* 000 is the number of the bootstrap samplings in the non-parametric tests.

Frequency band Regions (AAL3 atlas) *𝛿𝑧 𝑝*-value *𝑞*-value

Right Middle occipital gyrus 2.363 2.400E-04 1.074E-03 Left Middle temporal gyrus 2.275 0.000E+00 0.000E+00 Left SupraMarginal gyrus 2.272 6.000E-05 8.057E-04 Left Angular gyrus 2.218 1.200E-04 8.847E-04 Left Rolandic operculum 2.167 6.000E-05 8.057E-04

delta-theta

alpha

beta

Left Superior temporal gyrus 2.160 6.000E-05 8.057E-04 Left Middle occipital gyrus 2.081 1.400E-04 8.847E-04 Right Superior occipital gyrus 2.073 6.000E-04 1.312E-03 Left Superior occipital gyrus 2.064 8.000E-05 8.356E-04 Left Heschls gyrus 2.061 8.000E-05 8.356E-04

Left Fusiform gyrus -1.170 0.000E+00 0.000E+00 Left Inferior temporal gyrus -1.051 2.200E-04 1.034E-02 Left Parahippocampal gyrus -0.961 5.600E-04 1.065E-02 Left Inferior occipital gyrus -0.933 8.600E-04 1.065E-02 Left Hippocampus -0.928 9.400E-04 1.065E-02 Left Amygdala -0.912 8.800E-04 1.065E-02 Right Fusiform gyrus -0.891 1.340E-03 1.065E-02 Right Temporal pole: superior temporal gyrus -0.862 7.000E-04 1.065E-02 Left Middle temporal gyrus -0.835 2.080E-03 1.450E-02 Left Lingual gyrus -0.827 7.800E-04 1.065E-02

Left Superior temporal gyrus -1.132 2.800E-04 7.708E-03 Left Heschls gyrus -1.076 1.600E-04 7.520E-03 Left Angular gyrus -1.048 5.000E-04 7.708E-03 Left Rolandic operculum -1.026 4.400E-04 7.708E-03 Left Parahippocampal gyrus -1.011 4.000E-05 3.760E-03 Left Insula -0.994 5.600E-04 7.708E-03 Right Heschls gyrus -0.953 8.200E-04 7.708E-03 Left SupraMarginal gyrus -0.951 7.600E-04 7.708E-03 Left Lenticular nucleus-Putamen -0.934 6.400E-04 7.708E-03 Left Inferior temporal gyrus -0.919 8.200E-04 7.708E-03
