# Supplementary material for: Neurophysiological trajectories in Alzheimer’s disease progression
Source: eLife. 2024 Mar 28;12:RP91044. doi: 10.7554/eLife.91044 (PMC10977971; doi:10.7554/eLife.91044)
Supplement: Supplementary file 9. [file elife-91044-supp9.docx]

**Pairs of stages with signiﬁcant weighted-mean differences (***𝑞***<0.05, FDR corrected) in the trajectories of delta-theta-, alpha-, and** **beta-band long-range synchrony in the SAC-EBMs (Figure 3B, F, J in the main text).** The *𝑝*-values of 0.000E+00 denote a value less than 1/50*,* 000, where 50*,* 000 is the number of bootstrap samplings.

**delta-theta**

Stages *𝑝*-value *𝑞*-value (4,1) 5.560E-03 1.787E-02

(5,1) 4.180E-03 1.710E-02 (6,1) 0.000E+00 0.000E+00 (6,2) 1.200E-04 1.200E-03 (6,3) 5.020E-03 1.738E-02 (6,4) 1.258E-02 2.979E-02 (7,1) 0.000E+00 0.000E+00 (7,2) 1.600E-04 1.200E-03 (7,3) 3.620E-03 1.629E-02 (7,4) 9.040E-03 2.542E-02 (8,1) 0.000E+00 0.000E+00 (8,2) 9.200E-04 5.400E-03 (8,3) 1.250E-02 2.979E-02 (9,1) 1.080E-03 5.400E-03 (9,2) 4.920E-03 1.738E-02

(10,1) 1.400E-04 1.200E-03 (10,2) 1.040E-03 5.400E-03 (10,3) 7.100E-03 2.130E-02 (10,4) 1.226E-02 2.979E-02

**alpha**

Stages *𝑝*-value *𝑞*-value (2,1) 4.000E-05 1.800E-04

(3,1) 0.000E+00 0.000E+00 (4,1) 0.000E+00 0.000E+00 (4,2) 2.320E-03 7.457E-03 (5,1) 0.000E+00 0.000E+00 (5,2) 4.000E-05 1.800E-04 (6,1) 0.000E+00 0.000E+00 (6,2) 1.000E-04 4.091E-04 (7,1) 0.000E+00 0.000E+00 (7,2) 1.200E-04 4.500E-04 (8,1) 0.000E+00 0.000E+00 (8,2) 1.980E-03 6.854E-03 (9,1) 0.000E+00 0.000E+00 (9,2) 8.940E-03 2.514E-02

(10,1) 0.000E+00 0.000E+00 (10,2) 2.660E-03 7.980E-03

**beta**

Stages *𝑝*-value *𝑞*-value (2,1) 0.000E+00 0.000E+00

(3,1) 0.000E+00 0.000E+00 (4,1) 0.000E+00 0.000E+00 (4,2) 8.560E-03 2.568E-02 (5,1) 0.000E+00 0.000E+00 (5,2) 5.200E-04 1.950E-03 (6,1) 0.000E+00 0.000E+00 (6,2) 6.000E-04 2.077E-03 (7,1) 0.000E+00 0.000E+00 (7,2) 2.000E-05 9.000E-05 (7,3) 1.390E-02 3.909E-02 (8,1) 0.000E+00 0.000E+00 (8,2) 3.400E-04 1.391E-03 (9,1) 2.000E-05 9.000E-05

(10,1) 0.000E+00 0.000E+00 (10,2) 2.440E-03 7.843E-03
