# Supplementary material for: Neurophysiological trajectories in Alzheimer’s disease progression
Source: eLife. 2024 Mar 28;12:RP91044. doi: 10.7554/eLife.91044 (PMC10977971; doi:10.7554/eLife.91044)
Supplement: Supplementary file 10. [file elife-91044-supp10.docx]

**Top** 10 **regions with signiﬁcant weighted-mean differences (***𝑞<* 0*.*05**, FDR corrected) in regional variations of long-range synchrony** **during preclinical stages (stages 5 vs 1) [Figure 3D, H, L in the main text].** The *𝑝*- and *𝑞*-values of 0.000E+00 denote a value less than 1/50*,* 000, where 50*,* 000 is the number of bootstrap samplings.

Frequency band Regions (AAL3 atlas) *𝛿𝑧 𝑝*-value *𝑞*-value

Left Thalamus -1.588 0.000E+00 0.000E+00 Left Rolandic operculum -1.558 0.000E+00 0.000E+00 Right Olfactory cortex -1.553 0.000E+00 0.000E+00 Left Fusiform gyrus -1.547 0.000E+00 0.000E+00 Right Amygdala -1.540 0.000E+00 0.000E+00

alpha

beta

Left SupraMarginal gyrus -1.527 0.000E+00 0.000E+00 Left Lenticular nucleus-Putamen -1.507 0.000E+00 0.000E+00 Left Lenticular nucleus-Pallidum -1.501 0.000E+00 0.000E+00 Right SupraMarginal gyrus -1.481 0.000E+00 0.000E+00 Right Parahippocampal gyrus -1.480 0.000E+00 0.000E+00

Left Inferior temporal gyrus -1.771 0.000E+00 0.000E+00 Right Middle temporal gyrus -1.711 0.000E+00 0.000E+00 Left Thalamus -1.709 0.000E+00 0.000E+00 Left Angular gyrus -1.707 0.000E+00 0.000E+00 Right Lenticular nucleus-Pallidum -1.688 0.000E+00 0.000E+00 Right Superior frontal gyrus-dorsolateral -1.676 0.000E+00 0.000E+00 Right Lenticular nucleus-Putamen -1.662 0.000E+00 0.000E+00 Left Fusiform gyrus -1.659 0.000E+00 0.000E+00 Left Lenticular nucleus-Pallidum -1.647 0.000E+00 0.000E+00 Left Hippocampus -1.644 0.000E+00 0.000E+00
