# Supplementary material for: Neurophysiological trajectories in Alzheimer’s disease progression
Source: eLife. 2024 Mar 28;12:RP91044. doi: 10.7554/eLife.91044 (PMC10977971; doi:10.7554/eLife.91044)
Supplement: Supplementary file 11. [file elife-91044-supp11.docx]

**Pairs of stages with signiﬁcant weighted-mean differences (***𝑞***<0.05, FDR corrected) in the trajectories of delta-theta-, alpha-, and** **beta-band local synchrony in the SAC-EBMs (Figure 4B, F, J in the main text).** The *𝑝*- and *𝑞*-values of 0.000E+00 denote a value less than 1/50*,* 000, where 50*,* 000 is the number of bootstrap samplings.

**delta-theta**

Stages *𝑝*-value *𝑞*-value (6,1) 0.000E+00 0.000E+00

(6,2) 0.000E+00 0.000E+00 (6,3) 1.000E-04 1.957E-04 (6,4) 8.400E-04 1.512E-03 (6,5) 1.262E-02 2.103E-02 (7,1) 0.000E+00 0.000E+00 (7,2) 0.000E+00 0.000E+00 (7,3) 0.000E+00 0.000E+00 (7,4) 0.000E+00 0.000E+00 (7,5) 0.000E+00 0.000E+00 (7,6) 6.140E-03 1.063E-02 (8,1) 0.000E+00 0.000E+00 (8,2) 0.000E+00 0.000E+00 (8,3) 0.000E+00 0.000E+00 (8,4) 0.000E+00 0.000E+00 (8,5) 0.000E+00 0.000E+00 (8,6) 2.448E-02 3.934E-02 (9,1) 0.000E+00 0.000E+00 (9,2) 0.000E+00 0.000E+00 (9,3) 0.000E+00 0.000E+00 (9,4) 2.000E-05 4.091E-05 (9,5) 1.800E-04 3.375E-04

(10,1) 0.000E+00 0.000E+00 (10,2) 0.000E+00 0.000E+00 (10,3) 0.000E+00 0.000E+00 (10,4) 0.000E+00 0.000E+00 (10,5) 0.000E+00 0.000E+00 (10,6) 2.000E-05 4.091E-05

**alpha**

Stages *𝑝*-value *𝑞*-value (3,1) 2.720E-03 5.322E-03

(3,2) 2.864E-02 4.341E-02 (4,1) 0.000E+00 0.000E+00 (4,2) 2.600E-04 5.850E-04 (5,1) 0.000E+00 0.000E+00 (5,2) 0.000E+00 0.000E+00 (5,3) 7.020E-03 1.215E-02 (6,1) 0.000E+00 0.000E+00 (6,2) 0.000E+00 0.000E+00 (6,3) 1.600E-04 3.789E-04 (6,4) 1.334E-02 2.144E-02 (7,1) 0.000E+00 0.000E+00 (7,2) 0.000E+00 0.000E+00 (7,3) 2.000E-05 5.625E-05 (7,4) 3.980E-03 7.463E-03 (8,1) 0.000E+00 0.000E+00 (8,2) 0.000E+00 0.000E+00 (8,3) 4.000E-05 1.059E-04 (8,4) 4.400E-03 7.920E-03 (9,1) 0.000E+00 0.000E+00 (9,2) 0.000E+00 0.000E+00 (9,3) 0.000E+00 0.000E+00 (9,4) 6.000E-05 1.500E-04 (9,5) 9.600E-04 2.057E-03 (9,6) 2.894E-02 4.341E-02

(10,1) 0.000E+00 0.000E+00 (10,2) 0.000E+00 0.000E+00 (10,3) 2.000E-05 5.625E-05 (10,4) 1.340E-03 2.741E-03 (10,5) 8.320E-03 1.387E-02

**beta**

Stages *𝑝*-value *𝑞*-value (2,1) 1.540E-03 3.150E-03

(3,1) 0.000E+00 0.000E+00 (4,1) 0.000E+00 0.000E+00 (4,2) 5.360E-03 8.933E-03 (5,1) 0.000E+00 0.000E+00 (5,2) 7.400E-04 1.586E-03 (6,1) 0.000E+00 0.000E+00 (6,2) 0.000E+00 0.000E+00 (6,3) 3.900E-03 6.750E-03 (6,4) 1.480E-02 2.352E-02 (7,1) 0.000E+00 0.000E+00 (7,2) 0.000E+00 0.000E+00 (7,3) 5.000E-04 1.305E-03 (7,4) 2.340E-03 4.387E-03 (7,5) 2.314E-02 3.471E-02 (8,1) 0.000E+00 0.000E+00 (8,2) 0.000E+00 0.000E+00 (8,3) 5.600E-04 1.305E-03 (8,4) 2.180E-03 4.265E-03 (8,5) 1.516E-02 2.352E-02 (9,1) 0.000E+00 0.000E+00 (9,2) 0.000E+00 0.000E+00 (9,3) 1.400E-04 3.937E-04 (9,4) 5.600E-04 1.305E-03 (9,5) 3.460E-03 6.228E-03

(10,1) 0.000E+00 0.000E+00 (10,2) 0.000E+00 0.000E+00 (10,3) 2.000E-05 6.429E-05 (10,4) 6.000E-05 1.800E-04 (10,5) 5.800E-04 1.305E-03
