# Supplementary material for: Neurophysiological trajectories in Alzheimer’s disease progression
Source: eLife. 2024 Mar 28;12:RP91044. doi: 10.7554/eLife.91044 (PMC10977971; doi:10.7554/eLife.91044)
Supplement: Supplementary file 12. [file elife-91044-supp12.docx]

**Top** 10 **regions with signiﬁcant weighted-mean differences (***𝑞<* 0*.*05**, FDR corrected) in regional variations of local synchrony during the** **preclinical stages (stages 6 vs 1) [Figure 4D, H, L in the main text]**. The *𝑝*- and *𝑞*-values of 0.000E+00 denote a value less than 1/50*,* 000, where 50*,* 000 is the number of bootstrap samplings.

Frequency band Regions (AAL3 atlas) *𝛿𝑧 𝑝*-value *𝑞*-value

Right Middle occipital gyrus 3.089 6.000E-05 9.246E-05 Left Middle temporal gyrus 2.859 4.000E-05 7.094E-05 Left Superior temporal gyrus 2.815 2.000E-05 4.372E-05 Right Heschls gyrus 2.780 2.000E-05 4.372E-05 Left Rolandic operculum 2.767 0.000E+00 0.000E+00

delta-theta

alpha

beta

Left SupraMarginal gyrus 2.729 4.000E-05 7.094E-05 Left Angular gyrus 2.726 2.000E-05 4.372E-05 Left Heschls gyrus 2.670 0.000E+00 0.000E+00 Left Parahippocampal gyrus 2.664 0.000E+00 0.000E+00 Left Insula 2.638 0.000E+00 0.000E+00

Left Fusiform gyrus -1.835 0.000E+00 0.000E+00 Left Inferior occipital gyrus -1.700 0.000E+00 0.000E+00 Right Fusiform gyrus -1.671 0.000E+00 0.000E+00 Left Lingual gyrus -1.670 0.000E+00 0.000E+00 Left Inferior temporal gyrus -1.596 0.000E+00 0.000E+00 Left Hippocampus -1.570 0.000E+00 0.000E+00 Right Inferior occipital gyrus -1.554 0.000E+00 0.000E+00 Right Parahippocampal gyrus -1.551 0.000E+00 0.000E+00 Left Parahippocampal gyrus -1.549 0.000E+00 0.000E+00 Right Calcarine ﬁssure and surrounding cortex -1.491 0.000E+00 0.000E+00

Left Superior temporal gyrus -2.357 0.000E+00 0.000E+00 Left Heschls gyrus -2.351 0.000E+00 0.000E+00 Left Rolandic operculum -2.258 0.000E+00 0.000E+00 Left Angular gyrus -2.253 0.000E+00 0.000E+00 Left Hippocampus -2.249 0.000E+00 0.000E+00 Left Parahippocampal gyrus -2.227 0.000E+00 0.000E+00 Left Insula -2.227 0.000E+00 0.000E+00 Right Heschls gyrus -2.192 0.000E+00 0.000E+00 Left Lenticular nucleus-Putamen -2.148 0.000E+00 0.000E+00 Right Hippocampus -2.120 0.000E+00 0.000E+00
